# Disease tolerance: a protective mechanism of lung infections

**DOI:** 10.3389/fcimb.2023.1037850

**Published:** 2023-05-03

**Authors:** Jianqiao Xu, Nan Xiao, Dongsheng Zhou, Lixin Xie

**Affiliations:** ^1^ College of Pulmonary & Critical Care Medicine, 8th Medical Center, Chinese PLA General Hospital, Beijing, China; ^2^ Medical School of Chinese PLA, Beijing, China; ^3^ State Key Laboratory of Pathogen and Biosecurity, Beijing Institute of Microbiology and Epidemiology, Beijing, China

**Keywords:** disease tolerance, lung infection, mechanism, immunoparalysis, metabolic adaptation

## Abstract

Resistance and tolerance are two important strategies employed by the host immune response to defend against pathogens. Multidrug-resistant bacteria affect the resistance mechanisms involved in pathogen clearance. Disease tolerance, defined as the ability to reduce the negative impact of infection on the host, might be a new research direction for the treatment of infections. The lungs are highly susceptible to infections and thus are important for understanding host tolerance and its precise mechanisms. This review focuses on the factors that induce lung disease tolerance, cell and molecular mechanisms involved in tissue damage control, and the relationship between disease tolerance and sepsis immunoparalysis. Understanding the exact mechanism of lung disease tolerance could allow better assessment of the immune status of patients and provide new ideas for the treatment of infections.

## 1 Introduction

Lung infections occur frequently and impose high economic and social costs worldwide. COVID-19 has been prevailing for nearly two years and has infected more than 500 million people across 218 countries, leading to more than 6 million deaths. In addition to viruses, a variety of bacteria and fungi can infect the respiratory tract and cause significant morbidity and mortality (Prina et al., 2015). Many studies on lung infections have focused on clearing the pathogen through the use of antibiotics and by inducing host resistance mechanisms. Due to the widespread use of antibiotics in recent years, drug-resistant bacteria pose a great challenge for clinical use. Resistance is the ability to reduce pathogen burden by responding to the immune system and downstream events (Ayres and Schneider, 2012). However, excessive inflammation can result in fatal tissue damage. In the past decade, disease tolerance has been identified as a distinct defense strategy, and is defined as the ability to reduce the negative impact of infection on host health without directly affecting the pathogen burden (Medzhitov et al., 2012; McCarville and Ayres, 2018). Disease tolerance is not immune tolerance, which involves the self/non-self discrimination of T cells, although the mechanisms of disease tolerance and immune tolerance are similar in certain disease.

As the primary site of gas exchange, the lungs are the most common target of infections. The understanding of disease tolerance in the lung holds promise because, unlike antibiotics and vaccines, it points to methods of treating infections that exert evolutionary pressures on pathogens (Ayres and Schneider, 2012; Crane et al., 2018). In this review, we will summarize the triggers of disease tolerance in the lung and focus on the cellular and molecular mechanisms underlying disease tolerance to infection. We also discuss the relationship between tolerance and immunoparalysis and provide clinicians with new ideas for assessing the immune status of patients.

## 2 The triggers of disease tolerance in lung

### 2.1 Innate host tolerance

Early local immune defenses in the respiratory tract involve specific mechanisms that promote disease tolerance in response to infections (Iwasaki et al., 2017). The airway epithelial cells and mucosal layer continually defend against infection at its earliest stages, averting leukocyte recruitment and the subsequent inflammatory response. However, the barrier function is weak because the respiratory epithelium is one of the primary tissue types affected by pulmonary infection, with dysfunction and degradation of the epithelial layer being the primary mechanisms of viral diseases (Crane et al., 2018). Furthermore, interactions between airway epithelial cells(AECs) and alveolar macrophages(AMs) also play an important role in disease tolerance. CD200 and TGF-β on the respiratory epithelium, as negative regulatory cues, constantly block the inflammatory pathways in alveolar macrophages and prevent the expression of inflammatory cytokines (Hussell and Bell, 2014). A subset of AMs attached to the alveolar wall contain gap junction channels with AECs, which can transmit immunosuppressive signals through synchronized Ca^2+^ signaling to AECs and macrophages, and reduce endotoxin-induced lung inflammation (Westphalen et al., 2014).

### 2.2 Pathogen induced tolerance

Studies have shown that some pathogens can induce host tolerance responses. Granuloma is a typical pathological feature of tuberculosis (TB) that can control and contain the spread of TB without directly reducing the pathogen burden. As a disease tolerance medium, granulomas maintain proinflammatory signaling in the core and anti-inflammatory signaling in the periphery (Marakalala et al., 2016), inhibiting excessive proinflammatory responses and lung parenchymal damage (Divangahi et al., 2018). A study on chronic recrudescing malaria infections in mice showed that a single malaria episode can induce host disease tolerance and minimize tissue damage, stress, and pathology during subsequent infections (Nahrendorf et al., 2021). In addition, helminth-induced type 2 immune responses can inhibit the proinflammatory factor IL-17 and drive macrophage activation *via* an alternative IL-4R signaling-dependent pathway, which contributes to both anti-inflammatory and direct wound repair processes (Yap and Gause, 2018).

### 2.3 Drug-induced tolerance

Several classes of antibiotics are beneficial for infections, which cannot be explained by their direct antibacterial activities alone. A group of anthracyclines at low doses is very effective in conferring protection against severe sepsis in mice. The mode of protection manifests at the level of DNA damage response and autophagy in the lung, which confers disease tolerance to severe infection occurring irrespectively of the pathogen burden (Figueiredo et al., 2013). Colaco et al. also reported that tetracycline antibiotics can protect against sepsis without affecting pathogen load. Tetracycline antibiotics perturb the electron transport chain (ETC) by inhibiting mitochondrial function in protein synthesis, thereby decreasing tissue damage in the lung (Colaço et al., 2021). Studies have demonstrated that beyond their antibacterial activity, these antibiotics may become important triggers for disease tolerance.

### 2.4 Endotoxin-induced tolerance

A typical endotoxin induces a proinflammatory response to counteract the growth and dissemination of gram-negative bacteria. Initial exposure to LPS can decrease responsiveness to LPS challenge, a condition known as LPS tolerance (Ayres and Schneider, 2012). Low-dose LPS inhalation in neonates offers protection against asthma development, which is attributable to dendritic cells (DC) glucocorticoid-induced tumor necrosis factor receptor ligand (GITRL) downregulation, and Treg-mediated T helper cell suppression (Ding et al., 2020). Pretreatment with low-dose LPS reduces the immunopathology of gram-negative and gram-positive bacterial infections in the lung (Sano et al., 2002; Koch et al., 2007; Natarajan et al., 2008). The first exposure to LPS activated the aryl hydrocarbon receptor (AhR) and the hepatic enzyme tryptophan 2,3-dioxygenase, downregulating early inflammation and contributing to host tolerance[22]. On the other hand, a clinical study induced immunotolerance in healthy males using intravenous endotoxin administration as a model for sepsis-induced immunoparalysis. They demonstrated that immunotolerant monocytes are characterized by a loss of metabolic plasticity, which impacts the antimicrobial functions of monocytes and increases the risk of secondary infection (Grondman et al., 2019).

## 3 Mechanisms establishing disease tolerance

**Figure 1:**
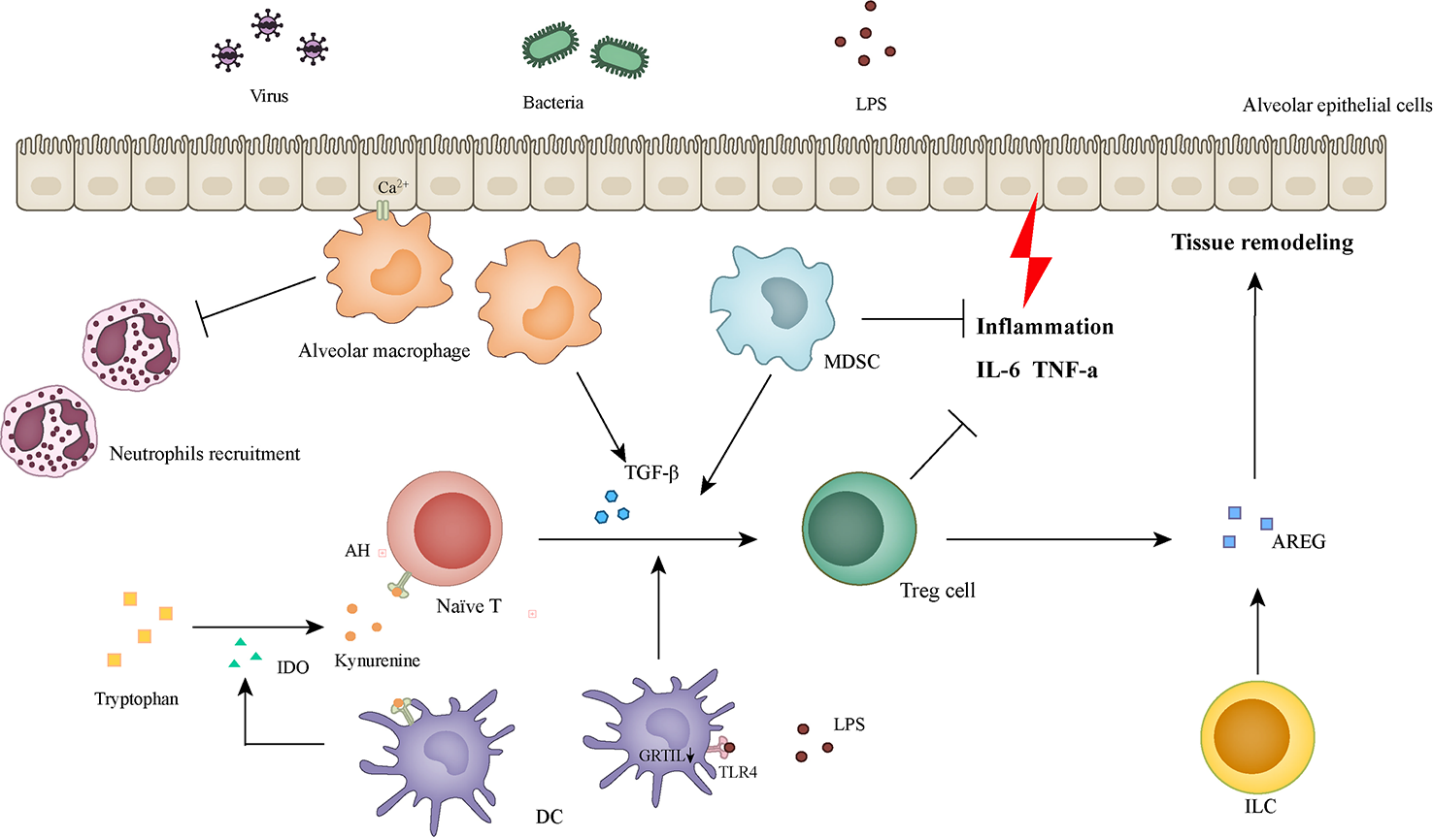
Cells and molecules involved in inflammatory regulation and tissue remodeling.

The specific mechanisms underlying disease tolerance remain unclear. The tolerance effect caused by different triggers varies according to the location or form of injury. However, most studies focused on the regulation of the host inflammatory response to control tissue damage (Soares et al., 2017). The relevant cellular and molecular mechanisms involve immune function regulation (**Figure 1**), metabolic adaptation, aging, obesity, and microecological changes. Finding commonalities is the focus of this part of the discussion.

### 3.1 Molecules in tissue damage control

Amphiregulin (AREG), a member of the epidermal growth factor (EGF) family, was originally described as an epithelial- and mesenchymal-derived factor. Recent studies have shown that AREG can be expressed by multiple populations of activated immune cells, such as mast cells, basophils, group 2 innate lymphoid cells (ILC2s), and macrophages (Zaiss et al., 2015; Minutti et al., 2019). The protective function of AREG has been demonstrated in multiple research (Monticelli et al., 2011; Jamieson et al., 2013; Soucy et al., 2018; Meng et al., 2020). AREG produced by acute lung injury(ALI) tissues and AMs directly inhibit TNF-α-induced apoptosis and transduction of caspase death signals in AECs as well as attenuate the severity of lung injury in mice (Meng et al., 2020). In a mouse model of influenza and *Legionella pneumophila* infection, AREG treatment decreased albumin levels in the bronchoalveolar lavage fluid (BALF) and pulmonary infiltrate and significantly decreased mortality without affecting viral and bacterial burdens (Jamieson et al., 2013). In addition, as the main product of innate lymphoid cells (ILCs), AREG restored lung function and promoted tissue remodeling in influenza virus-infected mice (Monticelli et al., 2011). These studies indicate that AREG plays an important role in promoting host tolerance by sustaining lung tissue integrity following infection-induced tissue damage.

Toll-Like Receptor 4 (TLR4) is a member of the TLR family, which comprises germline-encoded receptors expressed by cells of the innate immune system (Lu et al., 2008). As an important molecule for host resistance, TLR4 can lead to the production of proinflammatory cytokines and trigger inflammatory and immune responses when activated by bacterial LPS (Kuzmich et al., 2017). TLR4 is involved in the regulation of host tolerance. LPS preexposure downregulates the TLR4-dependent TRIF/IRF3/IFNβ pathway of DCs, leading to a skewed Treg cell population and offering protection against asthma development (Ding et al., 2020). TLR4 can also induce myeloid-derived suppressor cells (MDSCs) in the lungs to phagocytose apoptotic neutrophils during bacterial pneumonia and ultimately restore tissue homeostasis (Ray et al., 2013). In addition, to maintain this immunological balance, the proinflammatory signal of TLR can be attenuated by various negative regulatory mechanisms, such as intracellular inhibitors, membrane-bound suppressors, degradation of TLRs, and TLR-induced apoptosis (Liew et al., 2005).

Indolamine-2,3 dioxygenase (IDO) is a crucial intracellular enzyme in tryptophan (Trp) catabolism and related immune regulation (de Araújo et al., 2017). It can be induced by inflammation or T cell activation and plays a counter-regulatory role (Munn and Mellor, 2013). Upregulation of IDO has been found to suppress the immune response to tumors in many cancer types (Munn and Mellor, 2016; Lou et al., 2019). IDO links innate and adaptive immune responses to generate systemic tolerance by activating Tregs (Munn and Mellor, 2013). In a pulmonary model of paracoccidioidomycosis, IDO promotes the sustained synthesis of TGF-β and expansion of Tregs, which attenuates excessive inflammation and tissue damage and thus contributes to host fitness (de Araújo et al., 2017). However, the immunosuppressive effects of IDO may predispose the lungs to secondary bacterial infections, enhance morbidity, and slow recovery from influenza infection (van der Sluijs et al., 2006; Huang et al., 2013).

Aryl hydrocarbon receptor (AHR) is a ligand-operated transcription factor that is mainly expressed in barrier organs such as the skin, intestine, lung, and associated immune cells (Bock, 2020). AHR can be activated by endogenous Trp catabolites, such as kynurenine and downstream metabolites, which enhance tumor malignancy and suppress antitumor immunity (Sadik et al., 2020). The physiological effects of AHR activation include regulation of both innate and adaptive immunity. AHR integrates environmental, dietary, and microbial signals and modulates the inflammatory response mediated by LPS and TLR4 signaling (Rothhammer and Quintana, 2019). LPS-triggered tolerance, which protects mice against immunopathology in bacterial infections, requires the combined effects of AHR and IDO1 to downregulate early inflammatory gene expression (Bessede et al., 2014). AHR activation can lead to T cell differentiation into FoxP3^+^ T regulatory cells, further mediating immunosuppression and attenuating lung fibrosis (Mezrich et al., 2010; Takei et al., 2020). Tryptophan metabolism requires catalysis by IDO. AHR activation in DCs can also lead to IDO induction. The interaction between IDO and AHR further promotes Treg cell production (Mezrich et al., 2010). AHR can also control lung inflammation, airway hyperresponsiveness, and tissue remodeling in mice exposed to ozone by reducing IL-22 expression (Michaudel et al., 2020). Loss of AHR leads to the worsening of allergic asthma symptoms, such as airway inflammation, mucus production, airway hyperresponsiveness, and airway remodeling (Chang et al., 2020). Another study found that antibiotic treatment reduced the expression of AHR in the intestine, impairing the lung immune defense, which further confirmed the protective effect of AHR on lung function and disease severity (Tsay et al., 2019).

IL-22 is a member of the IL-10 cytokine family and is mainly produced by T helper (Th) 17 cells, γδT cells, NKT cells, and innate lymphoid cells (ILCs). It can promote tissue regeneration and repair after lung injury by inducing proliferation and inhibiting apoptosis of epithelial cells (Dudakov et al., 2015). The protective effect of IL-22 against pulmonary infection may be reflected in both resistance and tolerance. In a mouse model of secondary bacterial pneumonia after severe influenza infection, the presence of IL-22 reduced the inflammatory response, preserved epithelial barrier function, decreased the bacterial burden, and improved survival (Abood et al., 2019; Hebert et al., 2020). IL-22 can increase glycolysis in macrophages by downregulating genes involved in oxidative phosphorylation to enhance resistance to pneumococcal pneumonia (Trevejo-Nunez et al., 2019). However, in another study of pneumococcal superinfection following influenza infection in mice, treatment with exogenous IL-22 enhanced the lung barrier and reduced bacterial transmission but did not affect the bacterial burden (Barthelemy et al., 2018). IL-22 can also upregulate IFN-λ expression, inhibit neutrophil recruitment, and decrease epithelial damage (Broquet et al., 2020). IL-22 plays a role in disease tolerance mainly by maintaining the integrity of the lung tissue.

Other molecules also play a role in disease tolerance by reducing inflammatory responses. Leukotriene B4 is a type of lipoxygenase-derived lipid mediator that can suppress *in situ* monocyte-derived macrophage proliferation by promoting the production of IFN-α by pulmonary interstitial macrophages and controlling tissue damage in influenza A virus (IAV) infection (Pernet et al., 2019). Sphingosine1-phosphate (S1P) reduces the concentration of plasma cytokines and reactive oxygen species(ROS) in septic mice and promotes the chemotaxis and migration of ILCs to enhance the function of the endothelial barrier (Hemdan et al., 2016; Huang Y. et al., 2018). A20 (TNF alpha-induced protein 3) is a negative regulator of the antiviral immune response, and A20AEC-knockout mice show a better reduction in innate and adaptive immune responses, as well as lung damage than their wild-type littermates, although viral replication remains unaffected (Maelfait et al., 2016).

### 3.2 Cells in tissue damage control

Regulatory T (Treg) cells are a special CD4^+^ T cell subtype that specifically expresses the transcription factor Foxp3. Treg cells play an indispensable role in maintaining human immune tolerance and homeostasis and are an important component of the negative regulation of immune-mediated inflammation (Josefowicz et al., 2012; Sakaguchi et al., 2020). Treg cells promote disease tolerance in two ways. In the LPS-induced infection tolerance model, activated Treg cells reduced the inflammatory response by inhibiting the production of IL-6 and promoting the production of IL-10 by polymorphonuclear neutrophils (PMN) leading to suppression of the immune response (Lewkowicz et al., 2013). Similarly, during inflammation resolution, Treg cells can induce macrophages to produce IL-10 by secreting IL-13 and further activating the Vav-Rac1 pathway to promote phagocytosis of apoptotic cells (Proto et al., 2018). Treg cells also affect the function of other CD4^+^ T cells during chronic lung infections. In a study of long-term exposure to *Aspergillus fumigatus*, Treg cells which constrained the function of CD69^hi^CD103^lo^CD4^+^ tissue-resident memory T cells (TRM cells) were induced. The absence of Treg cells leads to deterioration of chronic lung inflammation and aggravation of fibrosis (Ichikawa et al., 2019). In addition to inhibiting lung tissue damage by other immune cells, another study has also found a special protective mechanism of Treg cells. They found that Treg cells can promote tissue repair following lung injury by producing AREG in response to the inflammatory mediators, IL-18 and IL-33, in the early stage of influenza virus infection, irrespective of their immunosuppressive effect. However, this process does not affect the viral burden or antiviral immune response (Arpaia et al., 2015). The tissue repair function of Tregs can be affected by Notch4 expression on its surface. Notch4 can inhibit AREG production and exacerbate inflammation, as observed in a cohort of patients with COVID-19 (Harb et al., 2021). In a model of LPS-induced lung injury, transcriptional profiles of type 2 alveolar epithelial (AT2) cells were analyzed in the presence or absence of Tregs. Researchers have found that Tregs affect a variety of functions of AT2 cells, especially by inhibiting the expression of IFN-γ and promoting the resolution of lung injury (Mock et al., 2020).

Innate lymphoid cells (ILCs) are a group of tissue-resident innate immune cells that share characteristics with T lymphocytes (Branzk et al., 2018). ILCs participate in a variety of immune pathways and cooperate with adaptive immune cells to regulate tissue inflammation (Vivier et al., 2018). Researchers have observed aggregation of ILCs in the lungs of mice after influenza virus infection. However, depletion of ILCs leads to airway epithelium damage and a decline in lung function. Through the analysis of the transcriptome of ILCs, it was found that a large number of AREG genes were enriched, which played an important role in tissue repair, and further confirmed the role of ILCs in maintaining the integrity of airway epithelium and homeostasis of the tissue environment (Monticelli et al., 2011). With the development of research, ILCs were divided into three subgroups (ILC1s, ILC2s, and ILC3s) according to their functional characteristics. ILC3s, which have functions similar to Th17 cells, are the main producers of IL-22 in the lung and are consequently important for lung tissue repair (Van Maele et al., 2014). Another study found that a decrease in ILCs and IL-22 can lead to exacerbation of pulmonary neutrophil inflammation, which is regulated by prostaglandin E2 (Felton et al., 2018). As a disease tolerance medium, tuberculous granulomas play a role in isolating pathogens, reducing bacterial transmission, and tissue damage. It was found that, in contrast to ILC1s and ILC2s, ILC3 is required for early protection against TB. The expansion of ILC3s and the production of IL-17 and IL-22 are key factors in the formation of granuloma-protective lymphoid follicles (Ardain et al., 2019). By analyzing the clinical data of patients with COVID-19, it was found that the ILCs were negatively correlated with the probability, duration of hospitalization, and severity of infection, which supported the importance of ILCs in disease tolerance (Silverstein et al., 2022).

Alveolar macrophages are sentinels in lung immunity. The function of alveolar macrophages can be influenced by the airway microenvironment and alters according to the tissue microenvironment (Hussell and Bell, 2014). Its role in repairing tissue damage and regulating inflammatory responses during lung infection cannot be ignored. Viral infection of alveolar macrophages was found in the autopsy of patients who died from COVID-19 and may be one of the important causes of cytokine storm and organ dysfunction (Wang et al., 2020). It has been found that repeated exposure to chlorine can lead to lung adaptation in mice, which is characterized by a significant reduction in the number of neutrophils and a decrease in airway hyperresponsiveness. Resident alveolar macrophages expressing Foxp3 mediate adaptation by enhancing phagocytosis and inhibiting inflammation due to the upregulation of the anti-inflammatory gene expression profile in these cells (Allard et al., 2019). Using real-time alveolar imaging in situ, researchers found that alveolar macrophages were connected to epithelial cells *via* connexin 43 (Cx43). Calcium ions transmit immunosuppressive signals between these two types of cells and inhibit the production of proinflammatory factors and neutrophil recruitment in LPS-induced inflammation (Westphalen et al., 2014). Alveolar macrophages expressing TGF-β and retinoic acid can induce naïve T cells to express Foxp3 and differentiate into Tregs, thus further controlling inflammation (Soroosh et al., 2013).

T lymphocytes are essential to defend against pathogens in adaptive immunity, especially in primary *Mycobacterium tuberculosis* (*Mtb*) infections. However, enhanced *Mtb* antigen-specific T cell responses cannot effectively eliminate tuberculosis but cause death in mice. Tzelepis et al. found that mitochondrial cyclophilin D (CypD) controls the expansion of activated T cells and limits disease severity, contributing to disease tolerance (Tzelepis et al., 2018). *Mtb*-specific T cells in granulomas can be affected by TGF-β and reduce the production of IFN-γ, resulting in local immunosuppression and affecting the clearance of tuberculosis (Gern et al., 2021). Another study has confirmed the role of T cells in tissue repair. They found that vaccinated BALB/c mice survived SchuS4 pneumonic tularemia, with significant numbers of highly virulent bacterial colonization. Limited tissue damage was observed, which was accompanied by increased AREG expression. Consequently, depletion of CD4^+^ T cells resulted in decreased AREG levels in BALF (Soucy et al., 2018); however, analyses of other CD4^+^ T cell subsets were not performed.

Dendritic cells (DCs) in the lungs comprise functionally distinct subsets. The aggregation and maturation of classical DCs in the lungs lead to an increase in neutrophil infiltration and Th1 cell activation after LPS stimulation, which aggravates pulmonary edema, acute pulmonary inflammation, and injury (Li et al., 2019). DCs not only promotes immunogenic responses as an important antigen-presenting cell but also maintain the tolerance of the lung mucosa. A microenvironment-adapted dendritic cell population, IFNAR1^hi^TNFR2^+^ conventional DC2 (iR2D2), was found to induce Treg cells in the lung at a steady state and prevent lung inflammation (Mansouri et al., 2020). IDO, as mentioned above, is secreted by DCs and promotes tryptophan metabolism. The metabolite (kynurenine) acts as a ligand of AHR to promote Treg generation from T cell precursors (Mezrich et al., 2010). GITRL is a key costimulatory molecule on the surface of DC that can inhibit Treg-mediated negative regulation by binding to its ligand on T cells. Endotoxin tolerance caused by low-dose LPS inhalation in neonatal mice can limit asthma development. Low-dose LPS induces downregulation of GITRL in dendritic cells, which promotes the differentiation of Treg cells and reduces the inflammatory response (Ding et al., 2020). Dendritic cell natural killer lectin group receptor-1(DNGR-1) in dendritic cells can inhibit neutrophil recruitment and control excessive inflammatory responses. However, DNGR-1 deficiency leads to an exacerbation of necrotizing pancreatitis and tissue damage without affecting the fungal burden (Del Fresno et al., 2018). CD1c^+^ DCs in COPD patients were insensitive to LPS and favor the generation of IL-10-secreting CD4^+^ T cells which play an immunosuppressive role hinder T-cell effector functions (Tsoumakidou et al., 2014), this tolerance mechanism may enable long-term pathogen colonization in patients with COPD.

Other cells may also be involved in the mechanism of tolerance to lung disease. Myeloid-derived suppressor cells (MDSCs) have a strong immunosuppressive ability and play an important role in immune regulation in cancer and chronic inflammatory diseases (Ray et al., 2013). In a *Klebsiella pneumoniae*-infected mouse model, monocyte MDSCs played a key role in reducing bacterial load and controlling lung injury by continuously producing IL-10 (Peñaloza et al., 2019). In the process of tumor immunity, MDSCs also can induce Treg cell differentiation and infiltration (Nagaraj et al., 2013). The role of NK cells in lung disease tolerance is still lacking; however, in helminth-infected mice, a large number of NK cells are recruited into the small intestine and initiate a type 1 immune response. The depletion of NK cells does not affect the worm load but increases vascular damage (Gentile et al., 2020).

Alveolar macrophages receive immunosuppressive signals from epithelial cells through Ca^2+^ signaling and inhibit the recruitment of neutrophils. Alveolar macrophages expressing TGF-β can induce naïve T cells to differentiate into Tregs. IDO secreted by DCs promotes tryptophan metabolism, and the metabolite (kynurenine) acts as a ligand of AHR to promote naïve T cells differentiation to Treg cells. Low-dose LPS induces the downregulation of GITRL in dendritic cells, which can also promote the differentiation of Treg cells. MDSC has a strong immunosuppressive ability and can also induce Treg cell differentiation and infiltration. In addition to inhibiting the lung tissue damage caused by inflammation, Treg cells can also promote tissue repair by producing AREG. AREG can also be generated by activated ILCs. IDO: indolamine-2,3 dioxygenase; DCs: dendritic cells; GITRL: glucocorticoid-induced tumor necrosis factor receptor ligand; MDSC: myeloid-derived suppressor cell; AREG: amphiregulin; ILCs: Innate lymphoid cells.

### 3.3 Metabolic adaptation

Activation of the immune system involves increased metabolic demands; therefore, the disease state is usually characterized by low energy consumption. Infection can lead to various symptoms including fever, fatigue, and anorexia. These symptoms may promote disease tolerance during metabolism (Medzhitov et al., 2012). Studies have found that, after inoculation with higher doses of *E. coli*, mice cultured at 22°C have better body temperature management and immunological consumption to ensure lower mortality. Simultaneously, the mice fasted the next day, recovered their body temperature and weight faster, and had reduced mortality without affecting the bacterial burden. The results show that energy-saving and low-metabolic states could promote tissue tolerance during bacterial infection (Ganeshan et al., 2019). Similarly, fasting metabolism has a protective effect on mice infected with bacteria because ketones produced by starvation can inhibit the production of ROS and reduce neuronal damage. However, inhibition of glucose utilization during viral infection leads to unfolded protein response(UPR)-mediated neuronal damage and increases mice mortality (Wang et al., 2016). Therefore, anorexia may be a self-protective strategy to induce tissue tolerance during bacterial infection.

Growth differentiation factor 15 (GDF15), a member of the TGF-β superfamily, is associated with many biological processes such as cancer, obesity, energy homeostasis, and body weight regulation (Baek and Eling, 2019). GDF15 plays an important role in tissue tolerance through metabolic regulation, which does not depend on resistance to pathogens or control of inflammatory response (Luan et al., 2019). In the process of acute inflammation caused by viruses or bacteria, GDF15 can promote the output of triglycerides in the liver, protect the heart from inflammatory damage, and reduce mortality. However, in the asthma model, Notch4-mediated GDF15 expression in Treg cells will activate ILC2 and promote inflammation. Simultaneously, this process limits the immunosuppressive function of Tregs and leads to the destruction of immune tolerance (Harb et al., 2020).

It has been widely reported that a dysregulated inflammatory response in sepsis leads to tissue and organ damage. Additionally, metabolic dysregulation plays an important role in organ dysfunction. In sepsis, heme produced by red blood cell lysis can inhibit liver glucose-6-phosphatase (G6Pase) through oxidative stress, thereby preventing liver gluconeogenesis and resulting in hypoglycemia. The ferritin H chain (FTH) can resist this inhibition and maintain blood glucose in the range of host survival (Weis et al., 2017). This interaction between host iron and glucose metabolism contributes to the disease tolerance of sepsis. The protective effect of FTH against *Mtb* infection was observed in FTH-knockout mice. A decrease in FTH levels leads to an excessive inflammatory response, which aggravates the disease. Abnormal iron metabolism was also observed in the lung tissues of patients with tuberculosis (Reddy et al., 2018). These studies have provided new insights into the role of iron metabolism in disease tolerance.

Disease tolerance plays a role in limiting the intensity of the inflammatory response and reducing tissue damage in acute infections, but it may also affect the clearance of pathogens during chronic infections. The prevalence of nosocomial *Klebsiella pneumoniae* (Kp) infections may be achieved by promoting the metabolic pathway of disease tolerance in the host. Kp induces glutamine decomposition and fatty acid oxidation, creating an oxidant-rich microenvironment that promotes the accumulation of MDSCs and M2 macrophages. This process induces disease tolerance by inhibiting inflammatory responses. However, Kp survives by upregulating the type VI secretion system to adapt to airway oxidants (Wong Fok Lung et al., 2022).

The immune metabolic crosstalk between specific pathogens and hosts has gradually been found by researchers. The typical pathogen Mycobacterium tuberculosis may reprogram host macrophages lipid metabolism to produce a phenotype rich in fatty acids to provide energy sources for themselves (Laval et al., 2021). Transcriptome analysis showed that AM increased the bacterial load through fatty acid oxidation, while IM limited the bacterial nutrient supply through glycolysis. These two different reactions constructed a balance between host immune resistance and tolerance (Huang L. et al., 2018).

### 3.4 Aging

Age was found to be a strong predictor of admission in patients with COVID-19, as well as disease severity and mortality, to some extent (Petrilli et al., 2020). The effects of aging on the tolerance of patients with COVID-19 have been reviewed, including the weakening of the UPR, mitochondrial dysfunction, and alteration of metabolic processes, which disrupt host resistance and disease tolerance (Mok et al., 2021).

The regulatory function of lung immune cells during inflammation decreases with age. 1A/1B-light chain-3 (LC3)-associated phagocytosis (LAP) is an important means of macrophage-mediated pathogen clearance and inhibits proinflammatory cytokine production during the innate immune response. Compared with young mice, elderly murine bone marrow-derived macrophages lack LAP, resulting in impaired bacterial killing and enhanced proinflammatory response after infection with *Streptococcus pneumoniae (* Inomata et al., 2020). In a mouse influenza infection model, researchers found an association between a decrease in the number of alveolar macrophages and impaired phagocytosis with age. At the same time, aging also affects the ability of alveolar macrophages to limit lung injury (Wong et al., 2017). Reduced tolerance of the elderly to infection leads to serious illnesses. In healthy adults, the proportion of neutrophils in BALF increases sometimes referred to as “inflammaging,” which leads to subclinical accumulation of proinflammatory factors and compromises disease tolerance during infections (Fulop et al., 2017). In elderly mice with LPS-induced lung injury, the level of extracellular superoxide dismutase (EC-SOD) enzyme decreased continuously and oxidative damage increased, leading to prolonged inflammation and pulmonary tissue damage (Starr et al., 2011).

The renewal and differentiation of type II alveolar epithelial cells (AEC2s) are important for maintaining alveolar integrity and lung barrier function. Researchers have found that the rates of bronchiolar club progenitor cell self-renewal and differentiation decrease with aging. In addition, the overall density of type I cells was decreased, and changes in genes related to proliferation and differentiation were found in the gene expression profile of epithelial cells, which is associated with age (Watson et al., 2020). Telomere-mediated senescence of AEC2s can lead to a serious decline in alveolar epithelial stem cell renewal and limit alveolar repair (Alder et al., 2015).

In conclusion, an uncontrolled inflammatory response and decreased alveolar regeneration may be important reasons for impaired disease tolerance in the elderly.

### 3.5 Obesity

Obesity has a significant influence on respiratory diseases. Obesity has marked effects on lung function, which is attributed to metabolic dysfunction and leads to increased susceptibility to infectious pathogens (Hornung et al., 2021). Obesity is a strong and independent determinant of severe COVID-19 infection and is an important risk factor for death from H1N1 influenza virus infection. Compared to the control group, obese mice showed a higher inflammatory response and more severe lung injury after H1N1 infection with no differences in the viral burden (Milner et al., 2015). Similar studies have found an increase in the number of infiltrated monocytes and chemokines, a significant decrease in airway epithelialization, and delayed wound repair in obese mice, which could be attributed to severe lung injury (O'Brien et al., 2012). Obesity causes macrophages and neutrophils to polarize towards M1 and N1 phenotypes, respectively, indicating increased activation states. Obesity also tends to increase neutrophil aggregation and expression of adhesion receptors in the lungs post LPS challenge (Manicone et al., 2016). Site-specific changes in the responsiveness of resident macrophages were found in obese rats upon LPS stimulation, while the TNF-α/IL-10 ratio was the highest in alveolar macrophages (Komegae et al., 2019). A clinical study found that the suppressive function of Tregs was impaired in obese patients, and the dysfunction was induced by obesity-related IL-18, which decreased the levels of FOXP3. Considering the important role of Tregs in regulating the pulmonary immune response, disease tolerance may be compromised in obese individuals.

### 3.6 Microbiome

Intestinal microbes affect the development and function of the immune system from the beginning of life. The lungs were originally considered sterile, however, novel innovations lead to the discovery of the lung microbiota. Studies have found that changes in microbiota types and total microbial content, known as ecological imbalances, may affect the health status of the respiratory tract (Whiteside et al., 2021). Mice pre-infected with *Staphylococcus aureus* showed significantly reduced lung injury following influenza virus infection. *Staphylococcus aureus* recruits peripheral monocytes into the lungs. Monocytes differentiate into M2 alveolar macrophages and secrete anti-inflammatory factors that inhibit inflammatory responses and enhance tissue tolerance (Wang et al., 2013). The nasal administration of *Lactobacillus rhamnose* can improve protection against respiratory syncytial virus infection, however, the immunomodulatory effects of various strains differ. Peptidoglycan *in Lactobacillus rhamnose* CRL1505 significantly improves the function of T cells and alveolar macrophages when infected with respiratory syncytial virus and pneumococcus, thereby increasing the secretion of IFN-γ, IL-10, and IFN-β, which can reduce bacterial colonization and limit tissue damage (Clua et al., 2017).

Bidirectional communication along the gut-lung axis also plays an important role in the regulation of disease tolerance. The gastrointestinal microbiota of mice in contact with soil was markedly different from that of mice housed in clean bedding. Soil-exposed mice sensitized to ovalbumin showed higher anti-inflammatory signals, and less severe pulmonary inflammation compared to the control group. The inflammatory state of the lungs also affects the composition of the intestinal microbiota (Ottman et al., 2019). *Escherichia coli* strains colonizing the mouse intestine can prevent muscle atrophy in *Burkholderia thailandensis* pneumonia, which alleviates weight loss caused by metabolic disorders and promotes tolerance to diverse diseases (Schieber et al., 2015).

### 3.7 The wisdom of pathogens

Many opportunistic pathogens use tolerance mechanisms to escape host immune clearance, resulting in chronic infection or long-term colonization. Granuloma is a sign of tuberculosis. If the pathogen is not eliminated, the host isolates the pathogen and prevents transmission by forming granulomas (Divangahi et al., 2018). In the research of tuberculosis animal models and human beings, it has been shown that the center of granuloma has an inflammatory environment, and the tissues around cheese body have a relatively anti-inflammatory feature, which enables tuberculosis bacteria to exist for a long time and lead to active disease attacks under specific circumstances (Marakalala et al., 2016). The multidrug resistant Klebsiella pneumoniae induces host specific metabolic reactions, such as the activation of glutamine decomposition and fatty acid oxidation which promotes the activation of M2 macrophages and MDSCs, and induces disease tolerance. In turn, bacteria adapt to oxidative stress to survive by up regulating their type VI secretion system (Wong Fok Lung et al., 2022).

## 4 Disease tolerance and immunoparalysis

Sepsis is a common cause of death due to infections and leads to dynamic changes in the immune status of the host. Cytokine storm, mediated by an excessive inflammatory response in the early stages of infection, can lead to tissue injury and organ dysfunction. Compensatory anti-inflammatory response syndrome (CARS) leads to persistent immune paralysis, which affects the clearance of pathogens and secondary infections. These two phenomena have a fatal impact on patients through diverse mechanisms. They cannot be distinguished as they involve a variety of positive and negative feedback mechanisms, as well as processes related to disease tolerance (Domínguez-Andrés et al., 2019).

Endotoxin tolerance is an important mechanism that protects hosts against excessive inflammatory damage after exposure to endotoxins, such as LPS. Endotoxin tolerance is also considered an important mechanism that leads to immunosuppression in sepsis because of its negative regulation of the inflammatory response (Kim et al., 2018; Grondman et al., 2019). Researchers found that endotoxin-induced immunotolerant monocytes lost metabolic plasticity, including cytokine production, oxidative burst, and microbial killing capacity when stimulated by LPS, which reflects sepsis-induced immunoparalysis (Grondman et al., 2019). Endoplasmic reticulum stress can restore the function of macrophages under endotoxin tolerance and activate the inflammatory response inhibited in the later stages of sepsis, thereby, eliminating pathogens (Kim et al., 2018). The activation of myeloid cells by LPS induces itaconate synthesis, which mediates the natural immune tolerance of human monocytes. These tolerizing effects are long-lasting, and inappropriate activation of these processes can lead to immunoparalysis. β-glucan-induced training immunity has the potential to enhance immune response and restore immune paralysis (van der Poll et al., 2017; Domínguez-Andrés et al., 2019).

Lung LPS tolerance significantly reduces TNF-α expression in lung lavage fluid, but it does not affect the number of neutrophils, myeloperoxidase activity, or expression of chemoattractants such as CXCL1 and CXCL2, indicating that it not only inhibits the inflammatory response but also preserves the antibacterial ability of the lung (Natarajan et al., 2008). However, only a few similar studies have been conducted. Therefore, the mechanism by which disease tolerance inhibits the inflammatory response without affecting pathogen clearance requires further study.

Immunoparalysis and disease tolerance are both states of immune hyporesponsiveness, and their mechanisms are complex. Many studies consider immunoparalysis to be a consequence of excessive tolerance in many studies. Whether there is an obvious boundary between the two definitions or to find a balance point, the host can not only maintain an efficient immune response but also be reasonably regulated to limit tissue damage.

## 5 Conclusion

Disease tolerance is one of the most important strategies of the host against infection, as it ameliorates both the pathogen-induced and inflammatory response-induced effects and limits damage to the host tissues. It occurs mainly by inhibiting the inflammatory response, promoting tissue repair, and regulating metabolism to maintain the integrity of the respiratory barrier and preserve lung function. Although many studies have provided a preliminary understanding of the mechanism of disease tolerance, it is still not known how to use the tolerance mechanism to effectively regulate the immune response. Disease tolerance is like a double-edged sword. It inhibits excessive inflammatory responses and protects the function of tissues and organs, but it also has the potential to affect pathogen clearance and induce immune paralysis. Therefore, accurately identifying the immune status of patients, limiting inflammatory injury by inducing tolerance, and changing the tolerance state to restore immune activity will be the focus of future research.

## Author contributions

The first draft of the manuscript was written by JX and NX, and the manuscript was revised by DZ and LX. All authors contributed to the article and approved the submitted version.
